# Environmental deprivation is associated with reward processing impairments and negative symptoms in schizophrenia

**DOI:** 10.1016/j.schres.2025.07.013

**Published:** 2025-07-31

**Authors:** Gregory P. Strauss, Lauren Luther, Ian M. Raugh, Shiyuan Zhang, Sierra A. Jarvis, Anna R. Knippenberg, Ada L. Hutcheson, Alex S. Cohen

**Affiliations:** a Department of Psychology, University of Georgia, Athens, GA, USA; b Department of Psychology, University of Alabama at Birmingham, Birmingham, AL, USA; c Department of Psychology, Louisiana State University, Baton Rouge, LA, USA

**Keywords:** Environment, Avolition, Anhedonia, Asociality, Alogia, Blunted affect

## Abstract

**Background:**

An environmental systems theory was recently proposed positing that negative symptoms of schizophrenia (SZ) are influenced by direct and indirect environmental factors that limit access to resources needed to complete social, recreational, and goal-directed activities.

**Method:**

Two studies were conducted to test key tenets of this theory. In each study, the Area Deprivation Index (ADI), an objective measure of resource deprivation, was calculated based on neighborhood-level geocoded data. Study 1 included healthy controls (CN: *n* = 78) and outpatients with schizophrenia (SZ: *n* = 114) who received clinical ratings of negative symptoms. Trait-level ADI scores were derived from home addresses at the time of testing. In Study 2, outpatients with SZ (*n* = 52) completed 1-week of digital phenotyping and a battery of laboratory-based reward processing tasks putatively related to the mechanisms underlying negative symptoms. ADI scores were derived at the state level using GPS coordinates at the time of ecological momentary assessment survey.

**Results:**

Across studies, greater resource deprivation measured via the ADI was associated with higher severity of negative symptoms and reward processing deficits.

**Conclusions:**

Findings indicate that environmental factors restricting access to resources are associated with negative symptoms and their underlying mechanisms in SZ. Environmental systems-focused interventions may offer a promising adjunctive treatment for negative symptoms when combined with pharmacological and psychosocial approaches.

## 1. Introduction

Schizophrenia (SZ) is a leading cause of functional disability worldwide (Charlson et al., 2018). Negative symptoms are a primary predictor of functional impairment and poor clinical outcomes (Piskulic et al., 2012; Strauss et al., 2010; Strauss et al., 2012), making them a critical treatment target. Unfortunately, pharmacological and psychosocial treatments have not proven efficacious (Fusar-Poli et al., 2015).

Current approaches to identifying treatment targets for negative symptoms have focused on person-level biological and psychological factors. Although these studies have produced valuable information regarding factors contributing to the onset and maintenance of negative symptoms, they have not resulted in the development of viable interventions. Is this because the most relevant person-level mechanisms have yet to be identified or adequately engaged by current treatments? Or are there non-person-level factors external to the individual that preclude potentially effective treatments from taking action?

It was recently posited that treatment progress for person-level pharmacological and psychosocial treatments is stalled because relevant environmental factors that pose barriers to accessing resources needed to perform social, recreational, and goal-directed activities have yet to be addressed (Strauss, 2021). Four environmental systems are proposed to interact with person-level factors to give rise to and maintain negative symptoms: the microsystem (the settings where individuals engage in activities, and the individuals within those settings who influence activities, roles, and social interactions), mesosystem (the connections among microsystems, such as individuals who bridge multiple settings or contexts), exosystem (indirect environments that influence resource availability, including crime and access to recreational facilities and transportation), and macrosystem (socio-cultural factors influencing emotional expression, emotional experience, and activities). There is preliminary support for associations between these environmental systems and negative symptoms. For example, greater negative symptom severity has been associated with: living in urban, impoverished, or under-stimulating environments and having smaller social networks, aberrant family social dynamics, greater local income inequality, and lower socio-economic status (Wing and Brown, 1968; Canive et al., 1995; Halford, 1991; Samele et al., 2001; van Os et al., 2002; Ove et al., 2004; Oshima et al., 2005; Thorup et al., 2006; Tibber et al., 2019). Similarly, self-report studies have indicated a link between negative symptoms and self-perceived environmental factors, such as resource deprivation within the microsystem (number of social and activity settings) and exosystem (economy, mass media, politics/laws, neighborhood crime, neighborhood aesthetics) (Strauss, 2023; Zhang et al., 2023). Subjective reports of environmental resource deprivation have also been associated with psychological processes putatively underlying negative symptoms (e.g., defeatist performance, asocial, and low pleasure beliefs) and partially mediate the association between these psychological processes and negative symptoms (Zhang et al., 2023). Thus, there is preliminary support for a link between negative symptoms and environmental resource reductions, with greater extant evidence for subjective than objective environmental indicators.

To extend this literature and examine the association between negative symptoms and objectively defined environmental factors, two studies were conducted. Both studies used an objective measure of environmental resource availability, the Area Deprivation Index (ADI; Kind and Buckingham, 2018). ADI scores are derived from census block (i.e., neighborhood) level information on poverty, education, housing, and employment. Greater ADI scores (higher resource deprivation) have been linked to increased probability of developing psychiatric diagnoses (e.g., Alzheimer’s dementia, suicidality, PTSD) and cognitive impairment (George et al., 2023; Gogniat et al., 2023; Zuelsdorff et al., 2020). In study 1, the ADI was calculated for participants’ home addresses, serving as a trait indicator of environmental resource deprivation. Trait ADI scores were correlated with interviewer-rated negative symptoms to test the hypothesized association with clinically-rated symptoms. In Study 2, participants completed 1-week of digital phenotyping, including both active (ecological momentary assessment surveys, ambulatory videos submitted to vocal and facial analyses) and passive (geolocation, accelerometry, ambient speech) validated measures of negative symptoms (Cohen et al., 2020, 2021; Cowan et al., 2022a, 2022b, 2023; Narkhede et al., 2022; Raugh et al., 2020, 2021; Strauss et al., 2022). State environmental resource deprivation scores were calculated by deriving ADI scores from GPS coordinate data obtained when individuals completed ecological momentary assessment surveys. State ADI scores were correlated with these digital phenotyping markers of negative symptoms to test the hypothesized association with state environmental factors. In Study 2, reward-processing measures assessing core RDoC positive valence system domains previously identified as key person-level mechanisms of negative symptoms in SZ were also administered, assessing: hedonic reactivity, reinforcement learning, effort-cost computation, and value representation. It was hypothesized that greater environmental resource deprivation would be associated with impairments on these reward-processing tasks, based on prior evidence linking environmental factors to other psychological processes related to negative symptoms (e.g., defeatist beliefs) (Zhang et al., 2023).

## 2. Methods

### 2.1. Participants

#### 2.1.1. Study 1

Participants included 114 outpatients with SZ and 78 healthy controls (CN). Groups did not differ on age, sex, race, or parental education; however, SZ had lower personal education (see Table 1). SZ were recruited from local community mental health centers and online or printed advertisements. Diagnoses were made via the Structured Clinical Interview for DSM-5 (SCID-5) (First et al., 2015a). SZ participant scores on the Brief Negative Symptom Scale (BNSS) suggest average levels of negative symptoms for chronic outpatients.

CN participants were recruited from the local community using printed and online advertisements. CN had no current major psychiatric diagnoses as determined via the SCID-5, no current schizophrenia-spectrum personality disorders as determine via the SCID-PD (First et al., 2015b), no lifetime history of psychotic or bipolar disorders, no family history of psychosis, and were not currently prescribed any psychotropic medications.

All participants reported being free from lifetime neurological disorders and had no history of substance use disorder diagnoses on the SCID. Written informed consent was obtained from all participants for a protocol approved by the University of Georgia Institutional Review Board and the study was performed in accordance with the ethical standards laid down in the 1964 Declaration of Helsinki and its later amendments.

#### 2.1.2. Study 2

Study 2 included 52 outpatients with SZ that were different cases from study 1 (see Table 1 for demographics). Recruitment and diagnostic procedures were identical to Study 1.

### 2.2. Procedures

#### 2.2.1. Study 1

Data collection occurred between 2018 and 2019. Participants completed a clinical interview, after which the BNSS was rated (Kirkpatrick et al., 2006, 2011).

#### 2.2.2. Study 2

Data collection occurred between 2018 and 2019. Study procedures occurred in 3 phases.

##### Phase 1:

In an initial laboratory visit, participants provided informed consent, completed clinical interviews, and received training on the use of the mEMA app (www.ilumivu.com) and study smartphone used for digital phenotyping data collection.

##### Phase 2:

Digital phenotyping data was collected for six days. Surveys were programmed to be presented via the mEMA app randomly within 90 min epochs from 9 AM to 9 PM. Surveys were prompted by a tone and were available for a 15 min window, after which they became disabled. The time between surveys was at least 18 min and no more than 180 min. Android study devices were used. Only apps required for study procedures (i.e., mEMA) were accessible to participants on the smartphone provided for the study.

##### Phase 3:

Participants completed reward processing measures and returned the smartphone, charger, and received financial compensation for their time in the amount of $20 per hour for time spent in the lab completing interviews or behavioral reward processing tasks and $1 per survey completed. They also received an $80 bonus for returning the equipment used in Phase 2.

### 2.3. Measures

#### 2.3.1. Study 1

SZ participants were rated on the BNSS following a brief interview using the recommended probes. The BNSS is a 13-item interview-based rating scale designed to assess the core domains identified in the NIMH Consensus Conference on Negative Symptoms (Kirkpatrick et al., 2006): anhedonia, avolition, asociality, alogia, and blunted affect. Each item is rated on a 0 (absent) to 6 (extremely severe) scale. Psychometric studies indicate that the BNSS has good internal consistency, temporal stability, inter-rater agreement, convergent validity, and discriminant validity (Galderisi et al., 2021; Weigel et al., 2023). Confirmatory factor analyses indicate that the optimal factor structure consists of a hierarchical model and a 5-factor model (Ahmed et al., 2019, 2022; Chang et al., 2021; Mucci et al., 2019; Strauss et al., 2018, 2019; Sun et al., 2021). As such, score calculations included: (1) 2 dimension score consisting of Motivation and Pleasure (MAP) and Diminished Expression (EXP); (2) 5 domain scores (anhedonia, avolition, asociality, alogia, and blunted affect).

ADI scores were derived using standard techniques from: https://www.neighborhoodatlas.medicine.wisc.edu/. The ADI is calculated from the freely available Neighborhood Atlas website funded through the National Institute of Health (Kind and Buckingham, 2018). Addresses were coded at the census block level. Census block groups (i.e., neighborhoods) created based on participant home addresses were linked to state and national ADI decile scores. Participants in Study 1 resided 131 unique census blocks, and those in Study 2 traversed through in 495 unique census blocks, all located within the southeastern United States. The composite ADI scores use 17 census-based indicators to characterize the environmental resource deprivation of a neighborhood relative to the participant’s state and nationally^1^.

**Fig. 1.**
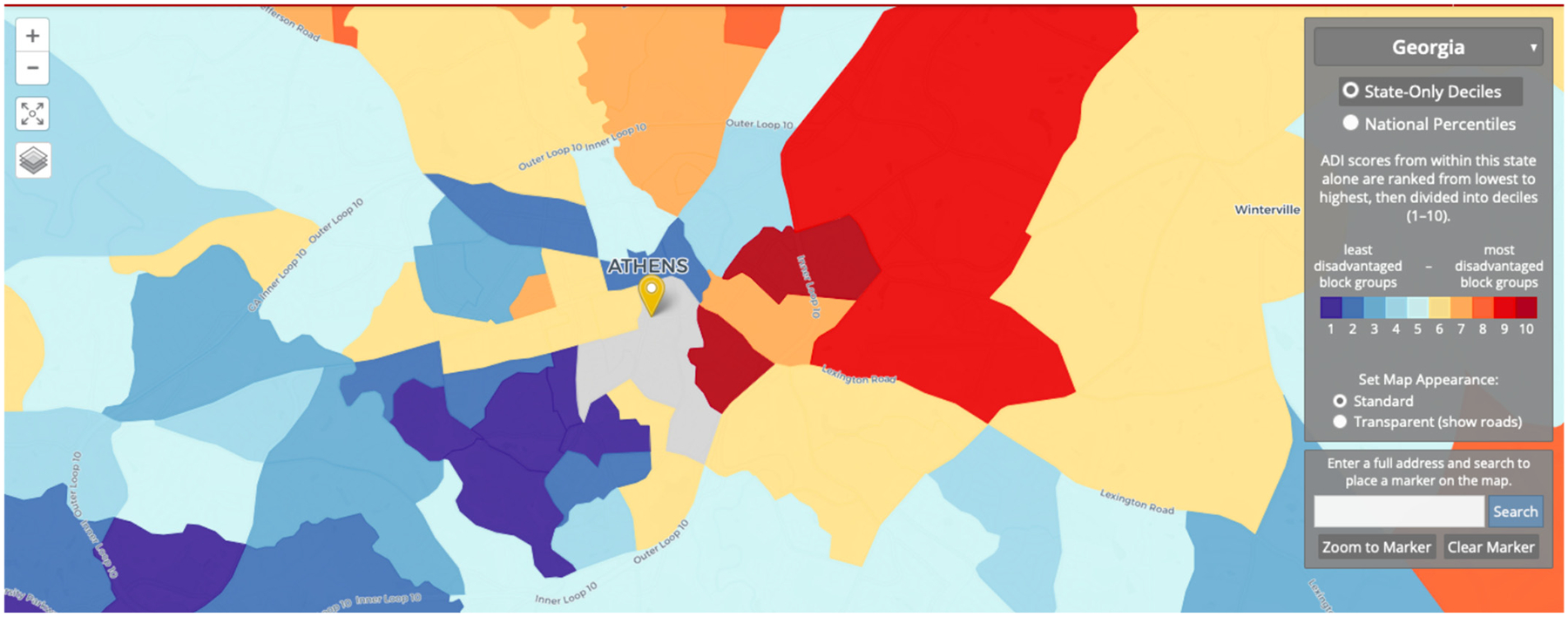
Area Deprivation Index map illustration. Note. The figure illustrates a geographic map displaying Area Deprivation Index scores in the Athens, GA area, where many participants were from. Red reflects areas with greater environmental impoverishment, whereas blue reflects areas with more advantage.

Trait ADI scores were calculated for Study 1 and reflect an ADI score paired with the participant’s home address at the time of the study. These ADI scores are stable factors, reflecting the most typical environmental exposure a participant receives (because home location is where they are likely to spend the most time on average). State ADI scores calculated for study 2 were based on the GPS location a person was at when they completed an EMA survey. The state ADI score thus reflects a dynamically changing influence of the environment on negative symptoms. Fig. 1 provides an example of ADI scores for locations around Athens, GA where many participants were recruited from.

#### 2.3.2. Study 2

EMA surveys assessed multiple psychological processes (e.g., emotional experience, emotion regulation). Only those germane to the current investigation focused on negative symptoms are described here. Mirroring procedures used by the BNSS, surveys included items required to assess both internal experience and behavioral components of anhedonia, avolition, and asociality. Behavioral components were assessed from context reports related to engagement in recreational, goal-directed, and social activities (Luther et al., 2023). Internal experience components were assessed via a combination of questions related to enjoyment, interest, and motivation for pleasurable, goal-directed, and social activities (see supplemental materials). At the end of each survey, participants were asked to self-record an ambulatory video of themselves giving a step by step description of their past hour while talking for at least 30 s (Cowan et al., 2022a; Cohen et al., 2013).

##### Ambulatory Video Processing:

FaceReader version 7.0, a commercially-available program, was used to measure facial expressions from ambulatory videos using validated procedures (Sun et al., 2021). A facial expression count metric was selected as a global measure of the frequency of facial expressions of emotion. Acoustic analysis was conducted using the Computerized Assessment of Affect from Natural Speech (CANS) (Cohen et al., 2009). Digital audio files were organized into “frames” and physical properties of speech were quantified (e.g., fundamental frequency, intensity). In this study, we evaluated data for indicies validated in prior studies related to pause length, sentiment, and word count (Cohen et al., 2020). To ensure adequate data for analysis, audio recordings with less than five utterances and video recordings with less than 10 % of frames were analyzable by Facereader were excluded.

##### Passive Digital Phenotyping:

Passive measures were collected from the smartphone (geolocation and accelerometry). Passive data (i.e., geolocation, accelerometry, VOX) was averaged within the 30-minute epoch around each active survey in order to pair passive and active data. All passive data collected from the phone sensors was encrypted and stored using unique identification codes on the Ilumivu servers, separate from identifying information, until it was downloaded by the research team onto a secure, password-protected server.

##### Geolocation:

Geolocation data was recorded every 10 min, when participants moved more than 10 m, and when an EMA survey was completed. Data was stored as GPS coordinates and changes in meters from the previous sample. Distance from home was calculated as the change in GPS coordinates at each sample from the participant’s home location. Participant home location was determined as the mean percentage of samples corresponding to the participant’s home, as endorsed by the participant. Geolocation has demonstrated good reliability and moderate convergent validity with negative symptoms (Parrish et al., 2020; Raugh et al., 2021).

##### Accelerometry:

Phone sensors were programmed to collect accelerometry values with each change in XYZ coordinate motion (every change in accelerometry being logged as a single instance), with separate values output for X, Y, and Z movement axes. Measurements related to average frequency of movement, vigor of movement, and variability in movement were calculated from phone accelerometry recording. Accelerometry has demonstrated good reliability and moderate convergent validity with clinically rated avolition (Strauss et al., 2022).

##### VOX:

Ambient sound vocal variables were collected for five second sampling periods every 10 min. During each sampling period, ambient sound was recorded at 16 kHZ. A vocal activity score (ACT) was calculated that reflects the number of frames in the audio sample in which voice was detected. No raw audio was recorded or retained. Higher ACT scores reflect a greater probability of social interaction occurring.

##### Reward Processing Tasks:

Participants completed four widely-used measures of reward processing: 1) a value representation task assessing the subjective value of a hypothetical future larger monetary reward versus a smaller immediate monetary reward (delay discounting task) (Kirby et al., 1999); 2) a hedonic reactivity or initial reward response task involving self-reported ratings of positive valence for pleasant images (Kirby et al., 1999; Strauss et al., 2017) from the International Affective Picture System (IAPS) (Lang and Bradley, 2007); 3) a probabilistic reinforcement learning task (Gold et al., 2012) assessing explicit learning from gains; 4) a probabilistic reinforcement learning task assessing implicit learning from gains (Pizzagalli et al., 2008); and 5) an effort-cost computation task—the EEfRT—where participants choose between completing a low effort button pressing task for a lower monetary reward value versus a high effort option for higher rewards (Treadway et al., 2009) (see Supplemental Materials for task details).

##### Defeatist Performance Beliefs Scale (DPB):

All participants completed the 15-item defeatist performance belief scale (Grant and Beck, 2009). This scale contains items that index overgeneralized beliefs about a person’s capacity to perform tasks.

### 2.4. Data analysis

#### 2.4.1. Study 1

One-way ANOVA was used to determine whether SZ and CN differed on ADI scores. Correlations were conducted between ADI scores tied to home address, along with ANOVAs for group comparisons. Bivariate correlations examined associations between ADI scores and BNSS dimensions/domains. SPSS Version 29 was used for all analyses.

#### 2.4.2. Study 2

Bivariate correlations examined associations between ADI scores based on GPS data and measures of reward processing and digital phenotyping indices of negative symptoms derived from active and passive data. Correlations were conducted between state GPS based ADI scores and their corresponding EMA scores, both of which were aggregated into 1 summary score to reflect the overall variance within a week of EMA recording. SPSS Version 29 was used for all analyses.

## 3. Results

### 3.1. Study 1

As hypothesized, SZ had significantly higher ADI scores than CN (see Table 2). Correlational analyses also indicated that greater negative symptom severity was associated with higher ADI scores (i.e., greater environmental resource deprivation) (see Table 3). This association was significant for the BNSS total score, MAP dimension, avolition domain, and alogia domain. Thus, both the diagnosis of SZ and negative symptoms were associated with greater environmental resource deprivation in Study 1.

### 3.2. Study 2

As hypothesized, higher trait ADI scores were associated with reward processing impairments, including lower hedonic reactivity, implicit reinforcement learning deficits, and explicit reinforcement learning deficits. Associations with effort-cost computation and value representation were nonsignificant (see Table 4). Notably, none of the reward processing variables were associated with antipsychotic medication status.

As hypothesized, higher state ADI scores, which reflect spending more time in resource impoverished areas, were associated with: 1) higher state anhedonia, avolition, and asociality measured via EMA; 2) less meters change, less distance from home, greater home time, and lower VOX ACT scores (less speech) measured via passive digital phenotyping; 3) greater pause length and fewer utterances on ambulatory videos; 4) higher defeatist performance beliefs measured via the trait DPB questionnaire (see Table 5).

## 4. Discussion

Two studies examined the association between an objectively geocoded measure of environmental resource deprivation (ADI: Area Deprivation Index), negative symptoms, and person-level mechanisms thought to underlie negative symptoms. Across studies, ADI scores were associated with greater severity of negative symptoms and person-level reward processing and psychological mechanisms of negative symptoms. Notably, associations were found when trait (based on home address) and state (based on GPS coordinates at the time of EMA survey) ADI scores were derived.

These findings support the environmental systems theory of negative symptoms proposed by Strauss (2021) and extend the literature in several important ways. First, most prior studies have relied on self-reported attributions of the environment, with studies finding that greater perceived resource deprivation in the microsystem and exosystem are associated with greater negative symptoms on clinical rating scales (Mehta et al., 2010; Strauss, 2023; Zhang et al., 2023). This study used an objective index that draws from standardized variables to capture 17 census-based indicators relevant to the exosystem that characterize the environmental resource deprivation of a neighborhood; these indicators have largely been unexamined in prior work in SZ but have been shown to be important for understanding how local environments may contribute to other forms of psychopathology (George et al., 2023; Gogniat et al., 2023; Zuelsdorff et al., 2020). Second, the use of both state and trait scores of environmental deprivation is novel, with use of EMA and passive mobile sensing affording examination of associations between microsystem environmental factors and negative symptoms in daily life with greater precision as digital phenotyping measures are less impacted by low resolution, recall demands, and halo effects than clinical rating scales (Cohen et al., 2021). Our results suggest that even state changes in one’s environment are associated with significant changes in multiple domains of negative symptoms measured both objectively and subjectively via digital phenotyping. The enhanced resolution also helps to elucidate how environmental deprivation influences more objective aspects of behavior related to negative symptoms, with our results indicating that greater state environmental deprivation is associated with less physical movement, more time at home, and reduced speech output.

Another key extension of prior work is demonstrating that environmental deprivation is associated with person-level factors (reward processing, defeatist beliefs) thought to be key mechanisms of negative symptoms. First, results demonstrated that living in a resource-impoverished area was associated with reduced reward processing in the domains of hedonic reactivity, implicit reinforcement learning, and explicit reinforcement learning. These findings are consistent with results from studies on the general population indicating associations between various forms of environmental deprivation (e.g., social deprivation, neighborhood poverty, prolonged exposure to under-stimulating environments) and impaired reward processing behavior or diminished activation of key reward circuits (e.g., ventral striatum to medial prefrontal cortex) (Hein et al., 2020; Marshall et al., 2018; Mehta et al., 2010). Early or chronic exposure to resource deprived environments may shape reward circuitry and the extent to which individuals respond to, learn about, and seek-out rewards in the service of goal-pursuit. Reductions in reward responsivity may even be adaptive in lower resource environments (Frankenhuis and Nettle, 2020) as rewards may be perceived as too personally costly and obtaining them may come at the expense of meeting immediate basic needs. Second, in an important extension of Zhang et al. (2023), our results suggest that greater objectively measured state environmental resource deprivation is associated with a psychosocial mechanism of negative symptoms, defeatist performance beliefs. It may be that resource poor environments provide fewer opportunities to counteract defeatist beliefs or that defeatist beliefs reduce a person’s likelihood of seeking opportunities that would facilitate engagement in more resource rich environments.

These findings also have important implications for psychosocial and structural interventions. The treatment of negative symptoms is currently stalled. These findings implicate a novel environmental target in resource deprivation that is not incorporated into current interventions for negative symptoms. Without changes in resource availability, it may be that the amount of change in negative symptoms that can be expected from negative symptom focused medications that augment appropriate biological mechanism is minimal. In other words, it may be necessary to increase resource availability and remove structural barriers within the direct and indirect environment in order for psychosocial and pharmacological treatments to achieve their effects after mechanistic engagement. Environmental systems focused psychotherapies may therefore be a necessary adjunct to pharmacological and psychosocial interventions for negative symptoms. These have proven efficacious for complex multifaceted disorders that have influences from biological, psychological, and environmental factors, such as conduct disorder (Henggeler, 1999). In this therapy, multiple environmental systems are comprehensively evaluated to understand their role in the patient’s symptoms. This serves as an individualized road-map for treatment, which includes using a mixture of person-, family, and systems-focused techniques. To address system level barriers, this treatment often involves working with family, neighbors, peers, school staff, and community organizations. Additionally, structural, policy level interventions may also be needed to address structural barriers that maintain negative symptoms. This includes structural interventions targeting social determinants like those facilitated by the World Health Organization and United Nations. Specific structural and environmental interventions that could be considered include: (1) community-level programs aimed at increasing access to recreational and vocational activities; (2) urban planning initiatives to improve walkability, reduce crime, and enhance social cohesion; (3) policy efforts that increase access to affordable transportation and healthcare; and (4) implementation of multisystemic therapy models that directly target barriers in the microsystem and exosystem. These interventions should be tailored to local needs and integrated with existing psychosocial treatments.

Certain limitations should be considered. First, these studies were cross-sectional, and causality can therefore not be determined. Second, only a small range of environmental processes were examined in relation to negative symptoms via the ADI score. Numerous others can be geocoded from address/GPS data or measured via interviews/questionnaires. A comprehensive battery of environmental measures is needed to identify additional environmental targets for negative symptom interventions. Additional measures which fit within the bioecosystem theoretical framework might include measures of social fragmentation (Ku et al., 2021), urbanicity (Grover et al., 2024), social cohesion, crime, street walkability, and other neighborhood characteristics (Solmi et al., 2020). Third, samples were geographically restricted within the Southeastern United States and did not comprehensively cover the range of urban and rural environments within the United States. Nationally representative samples are therefore needed. Fourth, these samples did not cover the full range of relevant ages and phases of illness. Developmental/illness effects are likely and should be studied systematically using a prospective longitudinal design with clinical high-risk, early psychosis, and chronic psychosis cases. Fifth, analyses could not address the length of time a participant lived at their home address and whether this duration impacts negative symptoms or reward processing.

Despite these limitations, several conclusions can be drawn. First, at a conceptual level, findings suggest that there should be an update regarding current views of how negative symptoms should be understood and treated. Current conceptual models of the mechanisms underlying negative symptoms focus on person-level factors such as reward processing, cortico-striatal circuitry, and defeatist performance beliefs. However, these models have yielded limited treatment breakthroughs, which may be due to the existence of environmental effects that have gone unstudied and untreated. Our findings point to a specific environmental target in resource deprivation. However, numerous other environmental variables could be relevant from the social, built, and physical environments. This includes macrosystem level factors such as structural racism and exosystem level factors such as politics, laws, and policies that limit access to resources needed to perform social, goal-directed, and recreational activities. If future studies replicate these associations between environmental processes and negative symptoms and extend them using prospective longitudinal studies, findings will suggest that novel environmental systems focused therapies and structural interventions should be attempted.

## Supplementary Material

1

## Figures and Tables

**Table 1:** Participant demographics in studies 1 and 2.

Study 1	SZ (*n* = 114)	CN (*n* = 78)	Test statistic	*p*
Age; M (SD)	41.6 (11.5)	39.3 (12.5)	F = 1.79	.18
% Male	36 %	32.1 %	χ^2^ = 0.31	.64
Personal education	13.3 (2.2)	15.2 (2.7)	F = 27.6	<.001
Parental education	13.5 (3.1)	14.0 (2.6)	F = 1.4	.24
Race			χ^2^ = 5.7	.18
African American	21.9 %	19.2 %		
Asian American	0.0 %	0.0 %		
Caucasian	71.1 %	64.1 %		
Hispanic/Latino	1.8 %	7.7 %		
Other	5.3 %	9.0 %		
BNSS Total	19.1 (12.7)	–	–	–

Study 2	SZ (n = 52)			
Age; M (SD)	39.9 (12.4)	–	–	–
% Male	34 %	–	–	–
Personal education	13.0 (2.2)	–	–	–
Parental education	13.8 (3.0)	–	–	–
Race				
African American	32.1 %	–	–	–
Asian American	0.0 %	–	–	–
Caucasian	54.7 %	–	–	–
Hispanic/Latino	3.8 %	–	–	–
Other	5.7 %	–	–	–
BNSS Total	16.1 (13.5)	–	–	–
EMA Survey Adherence	59 % (26 %)	–	–	–

*Note*. BNSS = Brief Negative Symptom Scale; CN = Control group; SZ = Schizophrenia group.

**Table 2:** Group differences in state and national ADI scores (Study 1).

	CN (n = 78)	SZ (n = 114)	F, *P*-value
ADI National	52.2 (24.2)	62.8 (22.4)	F = 9.8, *p* = .002

*Note*. ADI = Area Deprivation Index. ADI National scores range from 1 to 100, with higher scores reflecting more environmental deprivation relative to the United States Average.

**Table 3:** Correlations between area deprivation index scores and negative symptoms in schizophrenia (Study 1).

	r-Value
BNSS Anhedonia Domain	0.18
BNSS Asociality Domain	0.21
BNSS Avolition Domain	0.34**
BNSS Blunted Affect Domain	0.11
BNSS Alogia Domain	0.23*
BNSS MAP Dimension	0.30**
BNSS EXP Dimension	0.19
BNSS Total	0.25*

*Note*. BNSS = Brief Negative Symptom Scale (BNSS); MAP = motivation and pleasure dimension; EXP = diminished expression dimension.

* *p* < .05.

** *p* < .01.

**Table 4:** Correlations between reward processing measures and Area Deprivation Index scores in schizophrenia (Study 2).

	r-Value
Hedonic Reactivity	
Positive Emotion to Pleasant Stimuli	−0.07
Arousal to Pleasant Stimuli	−0.32*
Reinforcement Learning	
Explicit Learning from Gains	−0.39*
Implicit Learning Bias B3-B1	−0.36*
Value Representation	
Delay Discounting Geomean	−0.14
Effort-Cost Computation	
EEfRT % High Effort Choice High Magnitude	−0.19

*Note*. EEfRT = Effort Expenditure for Rewards Task.

* p < .05.

**Table 5:** Correlations between digital phenotyping measures and percentage time spent in Area Deprivation Index tracts measured via geolocation and geocoding in schizophrenia (Study 2).

	r-Value
Ecological Momentary Assessment	
EMA Anhedonia	0.37*
EMA Avolition	0.42**
EMA Asociality	0.37*
Passive Digital Phenotyping	
ACL mean	−0.12
GPS Meters from Home	−0.32*
VOX ACT	−0.36*
Ambulatory Videos	
Word Count	−0.44*
Pause Median	0.55**
Sentiment	−0.03
Facial Expression Count	−0.24

*Note*. ADI = Area deprivation Index; EMA = Ecological Momentary Assessment; GPS = global positioning system; VOX = measure of the presence of human speech detected amidst background environment.

* p < .05.

** p < .01.

## Footnotes

1 The 17 census-based indicators are as follows: % of population aged ≥25 with less than 9 years of education, % of population aged ≥25 with at least a high school diploma, % of employed persons aged ≥16 in white-collar occupations, median family income, income disparity (log of the ratio of households with ≥$50 K to ≤$10 K income), median home value, median gross rent, median monthly mortgage, % of owner-occupied housing units, % of civilian labor force population aged ≥16 unemployed, % of population below poverty level, % of population receiving public assistance income, % of households with no motor vehicle, % of households without a telephone, % of occupied housing units without complete kitchen facilities, % of occupied housing units without complete plumbing facilities, % of households with more than one person per room (crowding). See Kind and Buckingham (2018) for a full list of indicators used to derive the standard ADI score. Computing ADI scores involves a 5-step process: (1) Standardization (z-scores) of each variable to ensure comparability across indicators with different units or distributions. (2) Weighting via factor analysis: the ADI uses Singh’s original weighting scheme, derived through principal component analysis (PCA), based on a nationally representative sample. The weights assigned to each variable reflect its contribution to the shared construct of socioeconomic deprivation. (3) Computation of a composite index: The standardized and weighted scores are summed to form a base index for each block group; (4) Decile ranking: National ADI ranks all U.S. census block groups relative to one another (deciles 1–10; 10 = most deprived). State ADI ranks block groups within a given state (1–10) to enable intra-state comparisons. (5) Data outputs: provided as downloadable files for state- and notional-level ADI deciles, percentiles, and raw index scores. National ADI scores are reported in the manuscript and nearly identical results emerged with state-level decile scores.

## Appendix A. Supplementary data

Supplementary data to this article can be found online at https://doi.org/10.1016/j.schres.2025.07.013.
