# Supplementary material for: Environmental deprivation is associated with reward processing impairments and negative symptoms in schizophrenia
Source: Schizophr Res. Author manuscript; Available in PMC 2026 Jun 9. (PMC13249520; doi:10.1016/j.schres.2025.07.013)
Supplement: 1 [file NIHMS2180299-supplement-1.docx]

**Supplemental Materials for**

**ENVIRONMENTAL DEPRIVATION IS ASSOCIATED WITH NEGATIVE SYMPTOMS AND REWARD PROCESSING IMPAIRMENTS IN SCHIZOPHRENIA**

Gregory P. Strauss, Ph.D. ^1^*

Lauren Luther, Ph.D. ^1,2^

Ian M. Raugh, M.S. ^1^

Shiyuan Zhang, B.S. ^1^

Sierra A. Jarvis, B.S. ^1^

Anna R. Knippenberg, B.S. ^1^

Alex S. Cohen, Ph.D. ^3^

1. Department of Psychology, University of Georgia, Athens, GA, USA
2. Department of Psychology, University of Alabama at Birmingham, Birmingham, AL, USA
3. Department of Psychology, Louisiana State University, Baton Rouge, LA, USA

Correspondence concerning this article should be addressed to Gregory P. Strauss, Ph.D., Email: gstrauss@uga.edu. Phone: +1-706-542-0307. Fax: +1-706-542-3275. University of Georgia, Department of Psychology, 125 Baldwin St., Athens, GA 30602.

Reward Processing Task Descriptions

*Effort-cost computation.* The Effort Expenditure for Reward Task (EEfRT) (Treadway et al., 2009) assessed effort-cost computations. On each trial, participants are asked to choose between completing a low effort task (30 button presses in 7 seconds with their dominant hand index finger) for a lower reward value ($1) or a high effort option (100 button presses in 21 seconds with their nondominant hand pinky finger) for higher reward values ($1.24-$4.30). The probability of reward receipt associated with completed the selected task also varies across trials, ranging from high (88%), medium (50%), or low (12%). Following Barch et al. (2014), reward values were binned into four groups: low <$1.86), medium $1.96 to < $2.77), high $2.77 to <$3.58), and highest (>=$3.58) rewards. Participants are informed they will receive a portion of the money they earned during the task, but in reality, all received a $5.00 bonus. The primary dependent variable was the proportion of high effort tasks chosen in the high and highest reward and probability (88%) trials.

*Hedonic reactivity*. Pleasant, unpleasant, and neutral images from the International Affective Picture System (IAPS; Lang, Bradley, & Cuthbert, 2008) were used in an emotional experience paradigm that indexes hedonic reactivity (Strauss et al., 2018). Ninety pictures (30 in each condition) are presented. Participants are asked to rate from 1 to 9 how positive, negative, and calm/excited each image made them feel. The main dependent variables used were the average of the positive ratings for the pleasant images and the average arousal ratings (calm/excited) for the pleasant images.

*Value Representation.* Based on prior studies (Luther et al, 2020; Heerey et al., 2011), value representation was measured using Kirby et al’s (1999) delay discounting task. In this task, participants are asked across 27 trials to choose between a smaller, immediate monetary reward and a larger delayed reward. Larger delayed rewards range from small ($25-35), medium ($50-60), to large ($75-85). In this study, participants completed a computerized delay discounting task that involved hypothetical monetary amounts. Following Mazur (1987), a *k*-value for each choice was estimated. This value reflects the hyperbolic discounting parameter where participants are indifferent between the smaller immediate and later delayed rewards. To identify k-values for each reward size (small, medium, and large rewards), we calculated the geometric means (Kirby, 2000). The main dependent variable used in the current study was the average geomean score across the three reward sizes.

*Implicit Reinforcement Learning.* The Implicit Probabilistic Reward Task (Pizzagalli et al., 2008) was used to measure implicit reinforcement learning. The task involves completing a perceptual discrimination task the uses an asymmetrical reinforcement schedule in order to create a response bias for the stimulus that is rewarded more frequently. On each trial of the task, participants are shown one of two versions of a cartoon face and are asked to choose whether the face presented is one with the longer or shorter mouth. Correct responses receive a gain of $0.05 approximately 40% of the trials. Additionally, one of the two face stimuli is rewarded three times more often than the other. Feedback is not provided when incorrect responses are made. Participants completed 3 blocks of 30 trials. The main dependent variable in the current study was the learning from bias score where the number of correct responses on block 1 is subtracted from block 3 scores.

*Explicit Reinforcement Learning*. The Probabilistic Reinforcement Learning Task (PRLT; Gold et al., 2012) was used to assess the reinforcement learning domain of learning from gains. The PRLT is an explicit reinforcement learning task that assesses learning from both gains and losses. In the task, participants begin by completing an initial learning phase that that involves 160 trials where 4 sets of stimulus pairs of landscape images are displayed and probabilistically reinforced. Two stimulus pairs are associated with potentially gaining monetary rewards, and choosing the correct image is reinforced with a reward 90% of trials in one pair and 80% in the other pair. Choosing the incorrect image is not reinforced. The other pairs of stimuli are associated with avoiding potential monetary losses. Similarly, selecting the correct image results in feedback (90% or 80% of trials depending on pair) that the trial money is retained, while monetary loss occurs when the incorrect image is chosen. The percent correct in the most frequently reward gain stimulus pair (i.e., measuring an ability to learn from positive feedback during the highest rewarding condition) was the dependent variable in the current study based on prior work demonstrating that this condition had the strongest association with negative symptoms (Gold et al., 2012).

**Table S1**

*Ecological Momentary Assessment (EMA) Negative Symptom Survey Items*

| **Symptom** | **Scaling** | **Item text** |
| --- | --- | --- |
| Anhedonia internal experience | 0-100^1^ | How much are you enjoying the activity?  How much do you think you will enjoy that activity the next time you do it?  How much are you enjoying this social interaction?  How much do you think you will enjoy interacting with them next time? |
| Anhedonia behavior | 0 = Yes,  1 = No | What are you doing right now?  Recreation |
| Avolition internal experience | 0-100 | How interested are you in the activity? |
| Avolition behavior | 0 = Yes,  1 = No | What are you doing right now?^2^  Working/ Studying, Errands/ Housework, Exercising, Shopping, or Commuting/ Traveling |
| Asociality internal experience | 0-100 | How interested are you in this social interaction? |
| Asociality behavior | 0 = Yes,  1 = No | Who are you interacting with?^2^  Significant other, Family/ Roommates, or Friends |
| Negative symptom Composites | 0-200^1^ | Anhedonia internal experience * (1 + anhedonia behavior)  Avolition internal experience * (1 + avolition behavior)  Asociality internal experience * (1 + asociality behavior) |
| *Note*. * = Only assessed if emotion regulation endorsed. ^1^ = Average of all items. ^2^ = If any item selected, 1, otherwise, 0. | | |

**References**

Barch, D. M., Treadway, M. T., & Schoen, N. (2014). Effort, anhedonia, and function in schizophrenia: Reduced effort allocation predicts amotivation and functional impairment. *Journal of Abnormal Psychology, 123*(2), 387. https://doi.org/10.1037/a0036299

Gold, J. M., Waltz, J. A., Matveeva, T. M., Kasanova, Z., Strauss, G. P., Herbener, E. S., Collins, A. G. E., & Frank, M. J. (2012). Negative symptoms and the failure to represent the expected reward value of actions: Behavioral and computational modeling evidence. *Archives of General Psychiatry, 69*(2), 129-138. https://doi.org/10.1001/archgenpsychiatry.2011.141

Heerey, E. A., Matveeva, T. M., & Gold, J. M. (2011). Imagining the future: Degraded representations of future rewards and events in schizophrenia. *Journal of Abnormal Psychology, 120*(2), 483. https://doi.org/10.1037/a0022227

Kirby, K. N. (2000). Instructions for inferring discount rates from choices between immediate and delayed rewards. *Williams College, Williamstown, MA*. Unpublished manuscript.

Kirby, K. N., Petry, N. M., & Bickel, W. K. (1999). Heroin addicts have higher discount rates for delayed rewards than non-drug-using controls. *Journal of Experimental Psychology: General, 128*(1), 78. https://doi.org/10.1037/0096-3445.128.1.78

Lang, P. J., Bradley, M. M., & Cuthbert, B. N. (2005). International affective picture system (IAPS): Affective ratings of pictures and instruction manual (pp. A-8). Gainesville, FL: NIMH, Center for the Study of Emotion & Attention.

Luther, L., Fischer, M. W., Johnson-Kwochka, A. V., Minor, K. S., Holden, R., Lapish, C. L., McCormick, B., & Salyers, M. P. (2020). Mobile enhancement of motivation in schizophrenia: A pilot randomized controlled trial of a personalized text message intervention for motivation deficits. *Journal of Consulting and Clinical Psychology, 88*(10), 923. https://doi.org/10.1037/ccp0000509

Mazur, J. E. (2013). An adjusting procedure for studying delayed reinforcement. In *The effect of delay and of intervening events on reinforcement value* (pp. 55-73). Psychology Press.

Pizzagalli, D. A., Iosifescu, D., Hallett, L. A., Ratner, K. G., & Fava, M. (2008). Reduced hedonic capacity in major depressive disorder: Evidence from a probabilistic reward task. *Journal of Psychiatric Research, 43*(1), 76-87. https://doi.org/10.1016/j.jpsychires.2008.03.003

Strauss, G. P., Ruiz, I., Visser, K. H., Crespo, L. P., & Dickinson, E. K. (2018). Diminished hedonic response in neuroleptic-free youth at ultra high-risk for psychosis. *Schizophrenia Research: Cognition, 12*, 1-7. https://doi.org/10.1016/j.scog.2017.12.001

Treadway, M. T., Buckholtz, J. W., Schwartzman, A. N., Lambert, W. E., & Zald, D. H. (2009). Worth the ‘EEfRT’? The effort expenditure for rewards task as an objective measure of motivation and anhedonia. *PLOS ONE, 4*(8), e6598. https://doi.org/10.1371/journal.pone.0006598
